# A pilot study of endoscope-assisted MITLIF with fluoroscopy-guided technique: intraoperative objective and subjective evaluation of disc space preparation

**DOI:** 10.1186/s12893-022-01559-2

**Published:** 2022-03-23

**Authors:** Guang-Xun Lin, Chien-Min Chen, Gang Rui, Jin-Sung Kim

**Affiliations:** 1 Department of Orthopedics, The First Affiliated Hospital of Xiamen University, School of Medicine, Xiamen University, Xiamen, China; 2 Department of Neurosurgery, Seoul St. Mary’s Hospital, The Catholic University of Korea, 222 Banpo-daero, Seocho-gu, Seoul, 06591 Republic of Korea; 3 Division of Neurosurgery, Department of Surgery, Changhua Christian Hospital, Changhua, Taiwan; 4 Department of Leisure Industry Management, National Chin-Yi University of Technology, Taichung, Taiwan; 5 School of Medicine, Kaohsiung Medical University, Kaohsiung, Taiwan

**Keywords:** Degenerative lumbar spine, Disc space preparation, Endoscope assistance, Minimally invasive spinal surgery, Transforaminal lumbar interbody fusion

## Abstract

**Background:**

Adequate discectomy and endplate preparation are extremely crucial steps for spinal interbody fusion. Minimally invasive transforaminal lumbar interbody fusion MITLIF technique is safe and effective. However, concerns exist regarding sufficient disc space preparation from unilateral access. The purpose of this study, was to demonstrate our preliminary experience in objective and subjective evaluation of disc space preparation intraoperatively during endoscope-assisted MITLIF with fluoroscopy-guided, describing some of its possible advantages, and analyzing its safety and feasibility.

**Methods:**

From March 2018 to July 2019, three patients with degenerative spinal stenosis with radiculopathy and instability underwent endoscope-assisted MITLIF with fluoroscopy-guided. Patients’ demographic data, clinical parameters, subsidence, and fusion were collected.

**Results:**

Patients were successfully treated by endoscope-assisted MITLIF with fluoroscopy-guided at single-level or two-level. Symptoms improved postoperatively in all patients, and no complications occurred during follow-up. No cage subsidence was observed. At 6-month postoperatively, there was bony fusion observed on computed tomography in two patients.

**Conclusion:**

Endoscope-assisted MITLIF with fluoroscopy-guided is a safe and feasible technique to improve visualization during discectomy and endplate preparation objectively and subjectively, possibly increasing fusion rate and early time to fusion.

## Introduction

Minimally invasive transforaminal lumbar interbody fusion (MITLIF) to achieve disc space preparation and neural decompression via a unilateral approach with minimal disruption of only the single facet joint, and percutaneous pedicle screw fixation provides anteroposterior column support [1, 2]. Previously, the case series consistently reported acceptable fusion rates and good clinical outcomes for MITLIF [3, 4]. Adequate discectomy and endplate preparation are extremely crucial in spinal interbody fusion. Thus, it can be considered that a larger bone contact area between the graft and the vertebral bodies increase the likelihood of a successful interbody fusion. However, traditional instruments and techniques are still difficult to access specific areas of the intervertebral disc space, such as the contralateral posterior quadrant [5]. This is a challenging task, as the extent of discectomy cannot be visualized. To our knowledge, ideal disc space preparation involves the surgeon completely removing disc material and endplate cartilage, exposing the bleeding underlying endplate bone, and avoiding violations of the endplate [6].

We hypothesized that the aid of an endoscope and fluoroscopy-guided could improve intraoperative visualization, thus making it possible to prepare disc space sufficiently, while reducing injury to vertebral endplates, thereby beneficial for successful interbody fusion. This study demonstrates our preliminary experience of endoscope-assisted MITLIF (EA-MITLIF) with fluoroscopy-guided technique, describing some of its possible advantages, and analyzing its safety and feasibility.

## Methods

### Patient identification

This retrospective study has been approved by the Institutional Review Board (IRB) of the author’s institution, and all patients have obtained informed consent. From March 2018 to July 2019. All patients who were followed exceeded 12 months.

### Surgical technique

**Fig. 1:**
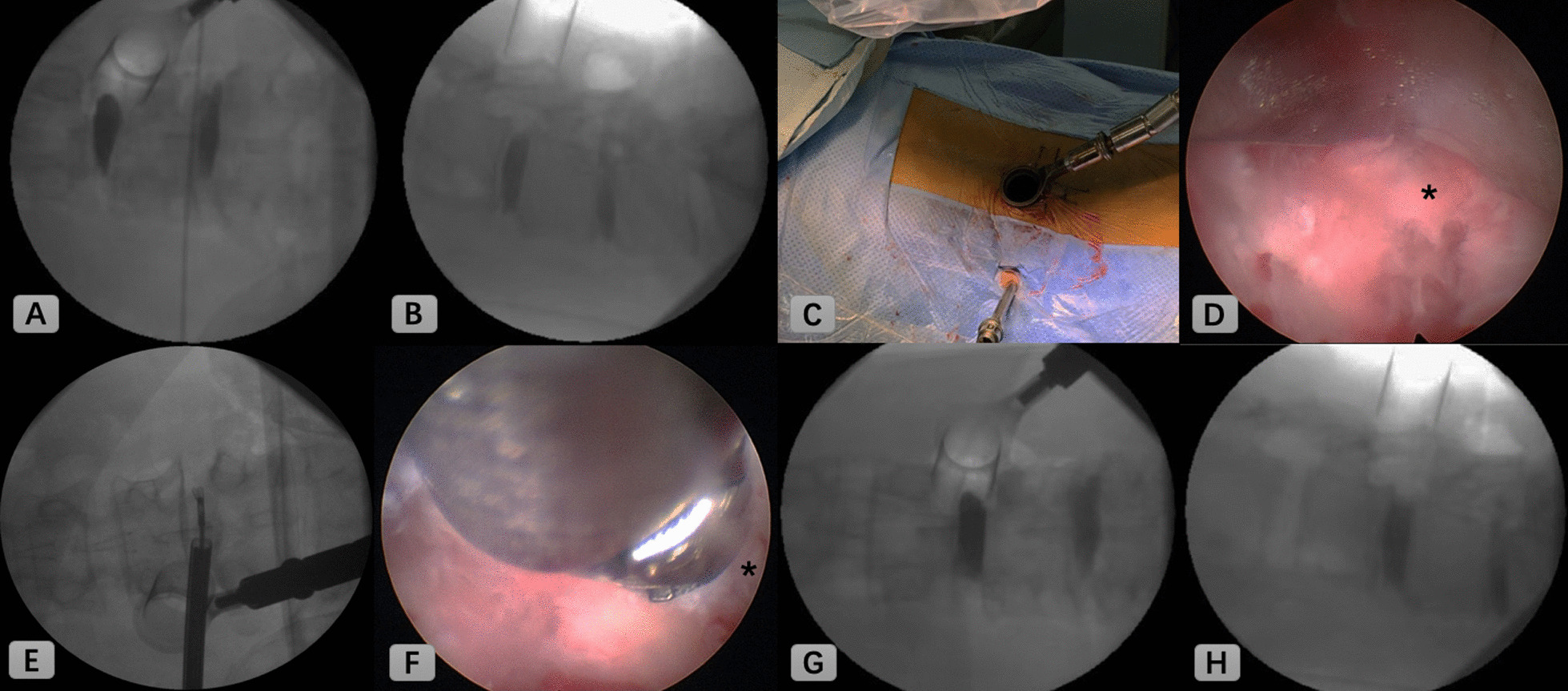
Intraoperative C-arm fluoroscopic and endoscopic images. **A**, **B** C-arm images were taken in anteroposterior and lateral views to confirm extent of discectomy. **C** An endoscope was introduced. **D** Endoscopic view of disc space. **E** Contralateral part of the disc was removed using endoscopic forceps. **F** Tip-control burr was shown removing endplate cartilage. **G**, **H** Adequate discectomy was confirmed when there was no gap between contrast medium and endplates. (*; endplate cartilage)

First, the patient was placed in a prone position on the Jackson table. Confirming index level under C-arm guidance, a 22 mm tube was introduced through the Wiltse approach. Visualization under microscopic view, unilateral partial laminectomy and facetectomy were performed, as well as bilateral decompression was conducted when necessary. Under C-arm guidance, preliminary disc space preparation was conducted in a blind fashion. After the blind preparation, a long needle is used to penetrate the radiopaque dye through the tubular retractor into disc space (Fig. 1A and B). To confirm the extent of disc space preparation using the anteroposterior and lateral C-arm images. Skin entry point 9–11 cm from the midline was made, then the guidewire, obturator, and final working sheath were inserted in accordance with the staged dilation procedure. A 25° endoscope (Vertebris Lumbar; RIWOspine, GmbH Knittlingen, Germany) was introduced to better visualize disc space (Fig. 1C and D). Endoscope assisted technique was more conductive to verify integrity and orientation of vertebral endplates during endplate preparation, also better access of the contralateral part of disc space (Fig. 1E). Using tip-control burr removal of endplate cartilage (Fig. 1F), exposing tiny bone bleeding underlying endplate bone. Following sufficient discectomy and endplates preparation [no gap between contrast medium and endplates (Fig. 1G and H)], morselized autograft bone was packed into the disc space. After then, insertion of a polyetheretherketone cage (Clydesdale, Medtronic Sofamor Danek, Memphis, Tennessee) filled with demineralized bone matrix mixed with local autograft bone. After insertion of the cage, we can visualize its position within disc space. After that, bilateral percutaneous pedicle screws fixation was conducted. The incision which introduced an endoscope can be used to insert the drainage.

### Outcome assessment

Patient demographic data, body mass index (BMI), bone mineral density (BMD), operative time, estimated blood loss, visual analogue scale (VAS), Oswestry Disability Index (ODI), cage subsidence, and bony fusion were evaluated.

Fusion and cage subsidence were observed on computed tomography (CT) scans postoperatively. Cage subsidence was considered as > 2 mm migration into either endplate. Bony fusion was defined as continuous presence of bridging trabecular bone and there was no gap between vertebral endplate and the cage. In consecutive coronal or sagittal views, observed connection of upper and lower endplates was also fused.

## Case presentation

### Case 1

**Fig. 2:**
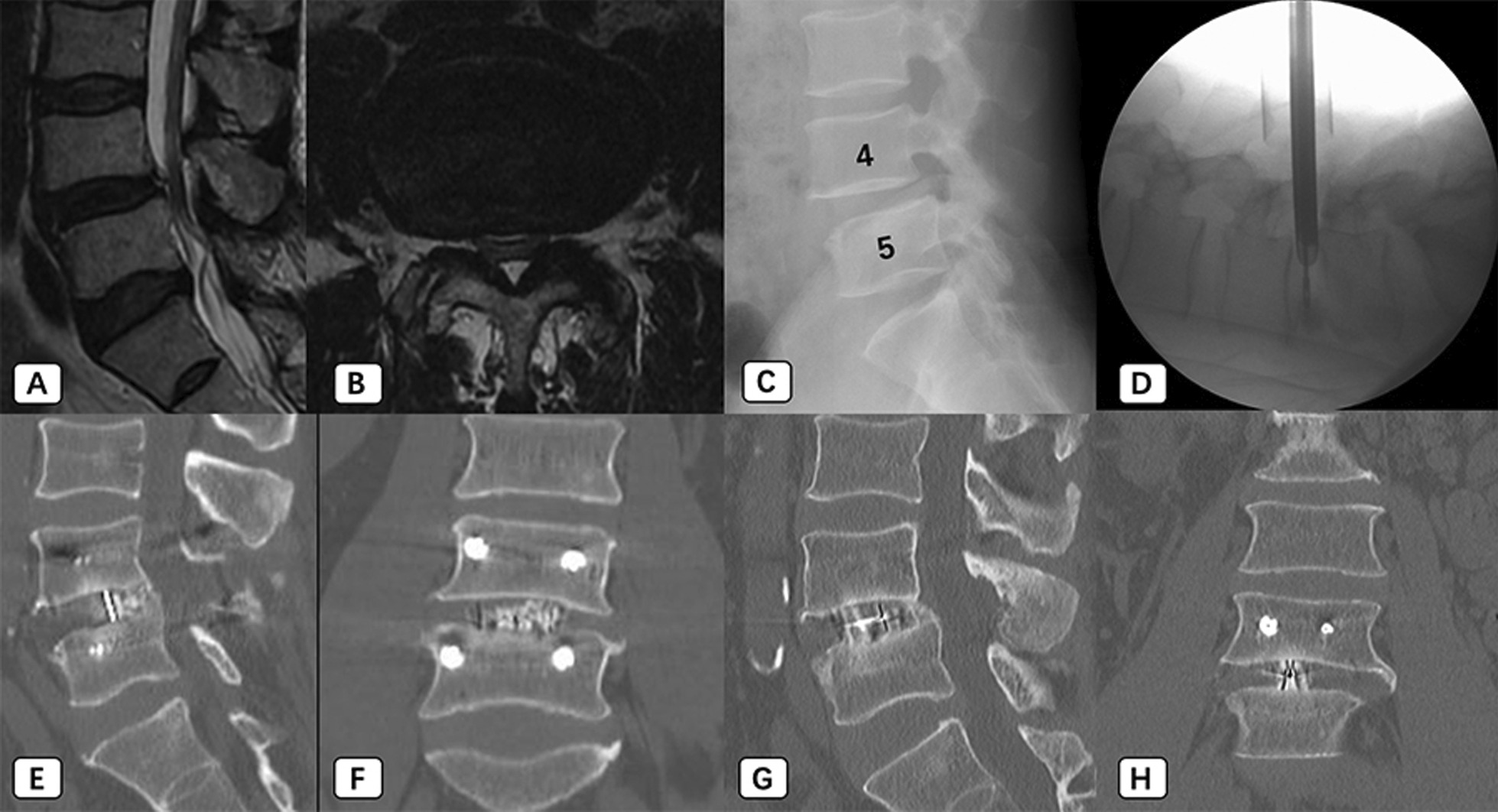
An age 50 male patient underwent endoscope-assisted minimally invasive transforaminal lumbar interbody fusion with fluoroscopy-guided technique at L4/5 level. **A**, **B** MR images show central stenosis with high-intensity zone at L4/5 level. **C** X-ray images show lumbar spondylolisthesis. **D** An endoscope was introduced to perform discectomy and endplate preparation. **E**–**H**, Bony fusion shows in sagittal and coronal computed tomography images at 6-month (**E** and **F**) and 20-month (**G** and **H**) postoperatively

An age 50 male patient (BMI: 22.4; BMD: − 1.6) complained about both buttocks and thigh (L5 dermatome) pain and paresthesia, which started 12 months ago. Magnetic resonance imaging (MRI) showed severe spinal stenosis at L4/5. Preoperative VAS for his back was 7, VAS for his leg was 7, and ODI was 43. After L4/5 EA-MITLIF with fluoroscopy-guided (unilateral decompression), the patient achieved excellent clinical improvement (at 12-month follow-up; VAS for back was 2, VAS for leg was 0, and ODI was 22). Estimated blood loss was 50 ml, operative duration was 195 min and hospital stays were 7 days. It showed a bony fusion at 6 months after surgery (Fig. 2). There were no perioperative or delayed complications.

### Case 2

**Fig. 3:**
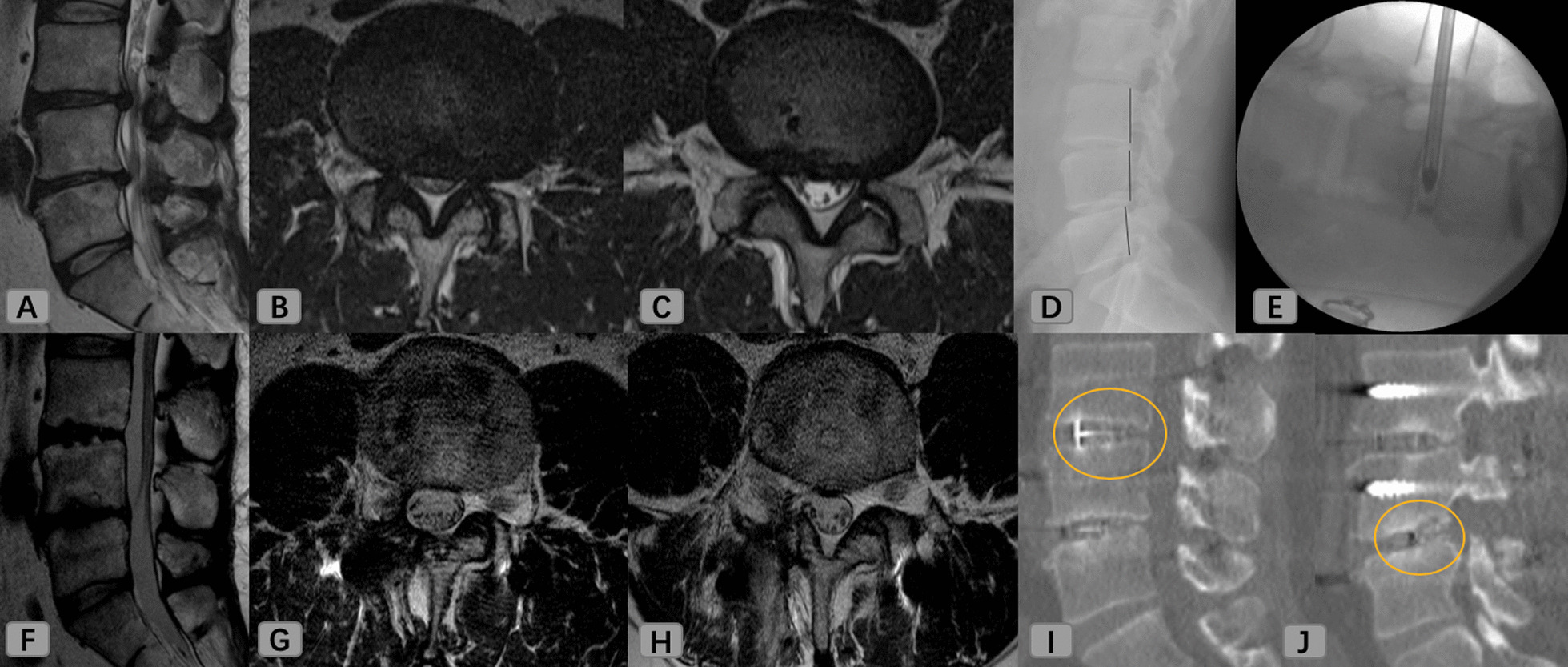
An age 49 male patient underwent endoscope-assisted minimally invasive transforaminal lumbar interbody fusion with fluoroscopy-guided technique at L3–4–5 level. **A**–**C** MR images show severe central stenosis at L3/4 (**B**) and L4/5 (**C**). **D** X-ray images show spondylolisthesis at L4/5. **E** An endoscope was introduced. **F**–**H** 1-year postoperative MR images. **I**, **J** Bony fusion (yellow circle) shows in coronal and sagittal computed tomography images at 6-month postoperatively

An age 49 male patient (BMI: 29; BMD: 0.4) presented with right leg radiating pain (L4, L5 dermatome), which started 9 months prior to his visit. MRI showed a severe spinal stenosis at L3/4 and L4/5. Preoperative VAS for his back was 6, VAS for his leg was 8, and ODI was 38. The surgeon performed L3/4/5 EA-MITLIF with fluoroscopy-guided (unilateral laminectomy with bilateral decompression). Estimated blood loss was 200 ml, operative duration was 330 min, and hospital stays were 8 days. At 6-month follow-up, the patient achieved bony fusion (Fig. 3). VAS for his back was 2, VAS for his leg was 2, and ODI was 18 at 12-month postoperatively. There were no complications reported during the follow-up.

### Case 3

**Fig. 4:**
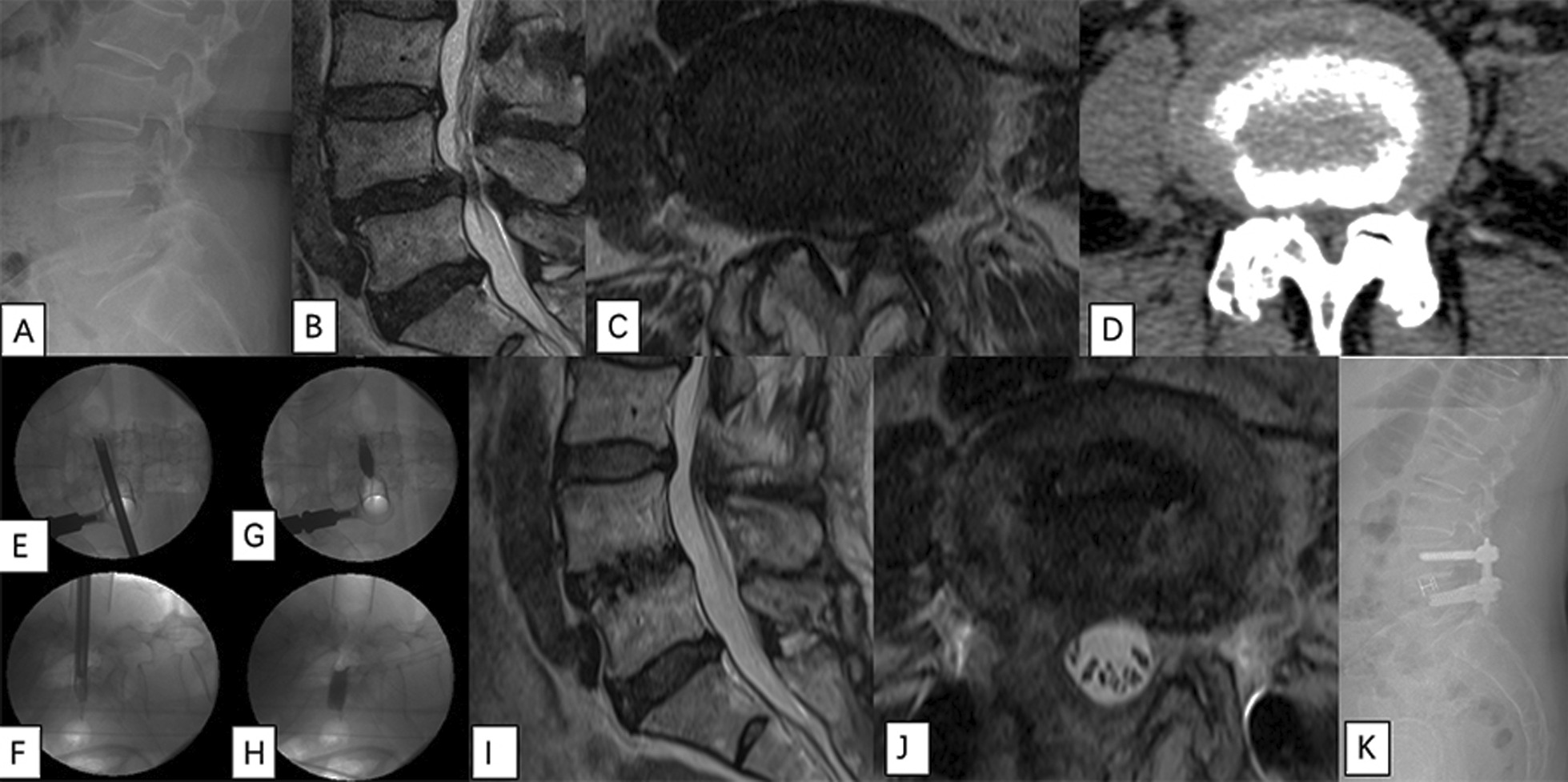
An age 67 female patient underwent endoscope-assisted minimally invasive transforaminal lumbar interbody fusion with fluoroscopy-guided technique at L4–5 level. **A** X-ray images show lumbar spondylolisthesis at L4–5. **B**, **C** MR images show severe central stenosis at L4–5. **D** CT image show severe stenosis at L4-5 and bilateral facet joint degeneration. **E**, **F** Disc space preparation using endoscope. **G**, **H** C-arm images were taken in anteroposterior and lateral views to confirm extent of discectomy. **I**, **J** 1-year postoperative MR images. **K** 1-year postoperative X-ray image

An age 67 female patient (BMI: 25.6; BMD: − 2.7) complained about both leg radiating pain (L5 dermatome) with neurologic claudication 100 m, which started 50 months ago. Magnetic resonance imaging (MRI) showed severe spinal stenosis at L4/5 (Fig. 4). Preoperative VAS for his back was 7, VAS for his leg was 9, and ODI was 44. After L4/5 EA-MITLIF with fluoroscopy-guided (unilateral laminectomy with bilateral decompression). Estimated blood loss was 200 ml, operative duration was 205 min and hospital stays were 7 days. The patient achieved excellent clinical improvement (at 12-month follow-up; VAS for back was 3, VAS for leg was 2, and ODI was 20). There were no perioperative or delayed complications.

## Discussion

MITLIF is a useful procedure in cases of degenerative lumbar diseases that improve clinical outcomes with good fusion rates [7, 8]. Disc space preparation is extremely important in any interbody fusion and is a crucial step in ensuring interbody fusion [9]. In theory, adequate discectomy and endplate preparation allow more bone graft material to be placed on a larger bed, thereby maximizing the fusion surface area. Nevertheless, according to previous literature, disc space preparation is not always ideal [10–12].

Although the MITLIF surgery has become more and more advanced, from the perspective of visualization and manipulation, it is a difficult task to prepare disc space through a tubular retractor with limited access from one side. Several cadaver studies have investigated that there are concerns about the adequate preparation of the endplate on the contralateral side of disc space [10, 11]. In particular, the contralateral dorsal quadrant is associated with lowest endplate preparation rate among all of the quadrants [5, 12]. Additionally, the highest amount of endplate damage occurred in the MITLIF at 48%, and there were no significant differences in endplate preparation rate between cranial and caudal endplates [10]. Additionally, compared with manual techniques, the discectomy device for disc space preparation has advantages in terms of short procedure duration and short instrument passage [12, 13]. A recent research found that there was significantly more disc material removed in the discectomy device group (48%) than in the manual discectomy group (38%), which was also the case in each quadrant (P < 0.05) [5]. However, in terms of endplate preparation, there were no significant differences (P > 0.05) between the device and manual discectomy groups [5]. Another recent cadaveric research found that there a higher disc space preparation rate using the CT-navigation guidance, especially in the anterior contralateral and posterior contralateral quadrants [14]. But, in terms of procedure duration, instrument passage times, and endplate violation rate, there were no significant differences (P > 0.05) between the two groups [14]. Our study was in line with above mentioned studies. Personally, the authors found that our EA-MITLIF with fluoroscopy-guided technique was particularly beneficial for visualizing disc space during disc removal and preparing the vertebral endplate. With the assistance of an endoscope, we could perform so that the annular and annulus remain intact; however, the periphery of the disc is removed up to the annulus to provide as much distraction and release as possible. We suggested that decortication on the endplate be performed, which would give the surgeon the best odds of better bony fusion development across disc space. Through our technique, it showed bony fusion at 6-month postoperatively. The early time for fusion is a merit of endoscopically assisted MITLIF compared to that observed with traditional MITLIF procedure.

It has been reported that cage migration or subsidence as related risk of performing MITLIF, could lead to non-union [15, 16]. Among them, early cage subsidence is directly related to intraoperative endplate preparation injury [17]. Endoscope visualization and intraoperative fluoroscope images could ensure intraoperative endplate violation, and also assess the correct position of the interbody cage. In this study, the authors describe how to apply an endoscope to preparation of the endplate and aggressive discectomy. There was no endplate violation and cage subsidence.

In summary, we propose an elaborate preparation of endplate with a measurement below which is worthy of notice. An objective evaluation is the measurement of the adequacy of intervertebral endplate on the fluoroscopic guidance and a subjective evaluation is the direct visualization of status quo of endplate on the endoscopic guidance.

This preliminary report has some shortcomings. The total number of patients was insufficient to reach a conclusion from current data, because only three patients underwent EA-MITLIF with fluoroscopy-guided. Also, the surgeon requires comprehensive knowledge and expertise in spinal endoscope and equipment. Too, operative duration seems to be lengthier than traditional MITLIF (compared to our previous study [18]; 1-level MITLIF took a mean of 182 min). In this study, 1-level procedure required 205 min and 195 min, and 2-level procedure was 330 min. Additionally, we minimize radiographic data because the authors do not think that endoscope-assisted procedure would affect lumbar lordosis or disc height.

No such research has been conducted on EA-MITLIF with fluoroscopy-guided so far, but since this pilot study has validated the technical aspects, we will introduce more cases. Based on authors’ immense experience of endoscopic spine surgery, this adoption of endoscope on MITLIF might not be genuinely novel, however, an effective and safe option to assess the competence of the disc space preparation intraoperatively. However, there may be additional cost-related claims, but no additional costs have been incurred because the introduction of these endoscopes has so far proved to be of any benefit. There is no basis for additional costs to be implemented in the country to which the author belongs.

Despite these shortcomings, based on findings of this study, EA-MITLIF with fluoroscopy-guided technique demonstrates safety and efficacy in a variety of adequate discectomy and endplate preparation, with present early duration to fusion and good clinical results. In the future, a randomized prospective study focused on a comparison of clinical and radiographic results between EA-MITLIF and traditional MITLIF will require larger sample sizes and longer follow-up duration.

## Conclusion

By the pilot study of endoscope-assisted MITLIF with fluoroscopy-guided technique, this is a technically feasible method to improve visualization during discectomy and endplate preparation, possibly increasing fusion rate and early duration to fusion and, ensuring integrity of the endplate.

## Data Availability

The datasets are presented within the manuscript.

## Abbreviations

BMD: Bone mineral density
BMI: Body mass index
CT: Computed tomography
EA-MITLIF: Endoscope-assisted minimally invasive transforaminal lumbar interbody fusion
ODI: Oswestry disability index
MITLIF: Minimally invasive transforaminal lumbar interbody fusion
MRI: Magnetic resonance imaging
VAS: Visual analogue scale
